# Modeling the Reflectance of the Lunar Regolith by a New Method Combining Monte Carlo Ray Tracing and Hapke's Model with Application to Chang'E-1 IIM Data

**DOI:** 10.1155/2014/457138

**Published:** 2014-01-12

**Authors:** Un-Hong Wong, Yunzhao Wu, Hon-Cheng Wong, Yanyan Liang, Zesheng Tang

**Affiliations:** ^1^Space Science Institute, Macau University of Science and Technology, Macao, China; ^2^School of Geographic and Oceanographic Sciences, Nanjing University, Nanjing 210093, China; ^3^Faculty of Information Technology, Macau University of Science and Technology, Macao, China

## Abstract

In this paper, we model the reflectance of the lunar regolith by a new method combining Monte Carlo ray tracing and Hapke's model. The existing modeling methods exploit either a radiative transfer model or a geometric optical model. However, the measured data from an Interference Imaging spectrometer (IIM) on an orbiter were affected not only by the composition of minerals but also by the environmental factors. These factors cannot be well addressed by a single model alone. Our method implemented Monte Carlo ray tracing for simulating the large-scale effects such as the reflection of topography of the lunar soil and Hapke's model for calculating the reflection intensity of the internal scattering effects of particles of the lunar soil. Therefore, both the large-scale and microscale effects are considered in our method, providing a more accurate modeling of the reflectance of the lunar regolith. Simulation results using the Lunar Soil Characterization Consortium (LSCC) data and Chang'E-1 elevation map show that our method is effective and useful. We have also applied our method to Chang'E-1 IIM data for removing the influence of lunar topography to the reflectance of the lunar soil and to generate more realistic visualizations of the lunar surface.

## 1. Introduction

Determination of the chemical compositions of planetary regolith is one of the most important objectives for planetary exploration, especially in estimating the mineral resources on a solar system body and choosing the landing site for landing probes on it. For this purpose, remote sensing has been used as an efficient approach. Modeling the reflectance of the lunar regolith is one of key issues in determining the chemical compositions of minerals with remotely obtained spectra. Radiative transfer models and geometrical optical models are commonly used for modeling the reflectance. While geometrical optical models [1–4] are used to investigate the ray's reflection/refraction between particles, radiative transfer models [5–12] focus on the reflectance intensity of different compositions of a mineral. Monte Carlo methods and ray tracing algorithms are widely used in simulating and analyzing the optically contrast structure of particles with complex shapes [1–4]. Lucey et al. [13–16] proposed a well approximation to find out the optical constants from a known reflectance spectrum using the inverse Hapkes model. Pieters and Hiroi [17] tested part of the lunar mineral samples taken back in the Apollo projects. The reflectance index of those tested sample is provided in the Lunar Soil Characterization Consortium (LSCC) data.

The existing methods for modeling the reflectance of the lunar regolith exploit either a radiative transfer model or a geometric optical model. However, the measured data from an Interference Imaging spectrometer (IIM) on an orbiter were affected not only by the composition of minerals but also by the environmental factors such as the reflection of the lunar topography, the position of light sources, and the position and orientation of the spectrometer. These factors cannot be well addressed by a single model alone. Due to these factors, the IIM data cannot be used directly with the existing models. Therefore, corrections and adjustments are needed [18]. Knowledge and experience are required to complete these corrections and adjustments, but the workload is very high.

In this paper, we try to consider these factors by modeling the reflectance of the lunar regolith with a new method combining Monte Carlo ray tracing for simulating the reflection of topography of the lunar soil and Hapke's model for calculating the reflection intensity of the internal scattering effects of particles of the lunar soil. Therefore, both large-scale and microscale effects are considered in our method, providing a more accurate modeling of the reflectance of the lunar regolith. To the best of the author's knowledge, it is the first attempt to propose a method considering the above mentioned effects at the same time.

**Figure 1:**
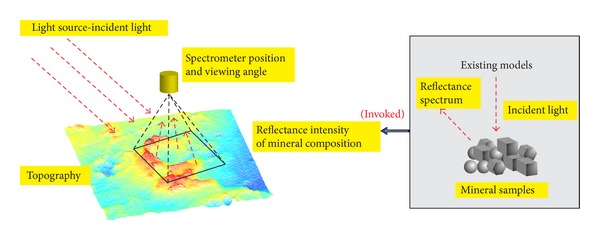
The schematic view of our method.

Figure 1 gives the schematic view of our method. Our method considers the reflectance attributes of the lunar soil, the terrain of the lunar surface, the incident light, and the position and viewing angle of the spectrometer and applies the existing reflection models to the large-scale scene of lunar surface. Our method simulates the scene when the spectrometer was measuring the reflectance spectrum and the reflected light of the specific categories of the lunar soil and the terrain captured by the spectrometer.

Our paper is organized as follows. A brief review of Hapke's model will be given in Section 2. In Section 3 we will explain some definitions and assumptions of the lunar environment and lunar soil, including some factors and considerations of the design of our method. The method and its simulation steps will be described in Section 4. In Section 5 we will provide the simulation results of the Apollo 16 landing-site using Chang'E 1 elevation map and the mineral information from the LSCC data. Two example applications of our method will be given in Section 6. Conclusions and future work will be provided in Section 7.

## 2. A Brief Review of Hapke's Radiative Transfer Model

Hapke's radiative transfer model was proposed by Hapke [6–12]. The equation we used in our simulation is shown as follows:

(1) $$\begin{array}{c} r_{c}=\frac{\omega }{4\left(\mu_{0}+\mu \right)}\left\{P\left(g\right)\left[1+B\left(g\right)\right]+H\left(\mu \right)H\left(\mu_{0}\right)-1\right\}, \end{array}$$

where the dimensionless quantity *r*_*c*_ is the radiance coefficient, the variables *μ* and *μ*_0_ are the cosines of the reflection and incidence angles, *g* is the phase angle, *B*(*g*) is the back scattering function which defines the increase in brightness of a rough surface with decreasing phase, *P*(*g*) is the single-particle phase function, and the *H*(*μ*) is the isotropic scattering function. Details of these functions and variables are described clearly in Lawrence and Lucey's paper [16].

**Figure 2:**
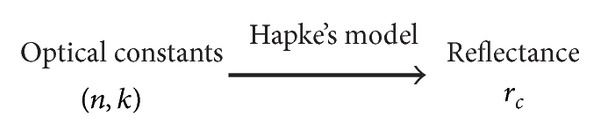
Hapke's model, reflectance can be calculated when optical constants/single scattering albedo are known.

One of the most important parts of (1) is the single-scattering albedo *ω*. Optical constants (*n* and *k*) are used in the calculation of *ω*. Hapke's model actually defines a formula between the optical constants and the radiance coefficients. Optical constants, also known as the complex indices of refraction, are used to predict the bidirectional reflectance of a particulate surface. If the optical constants of minerals in a mixture are known, then a reflectance spectrum can be calculated at arbitrary grain sizes [6].  Figure 2 shows the calculation of the reflectance by Hapke's model using optical constants as input.

**Figure 3:**
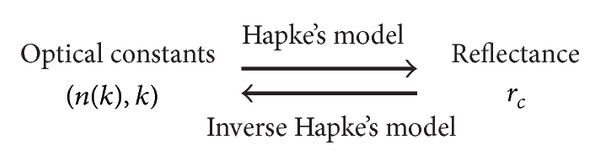
After the relationship between *n* and *k* are defined, optical constants can be found with inverse Hapke's model.

However, the optical constants of minerals are hard to be obtained. Lucey [13] defined the relationship between *n* and *k* via the Mg-number (Mg#—the ratio of Mg to Mg + Fe on a molecular basis, is defined as: Mg# = MgO/(MgO + FeO)). With Mg#, *n* can be represented as a function of *k*—*n*(*k*). Then the unknown numbers in both side of (1) are *r*_*c*_ and *k*. That means *k* can be calculated when *r*_*c*_ is known via inverse Hapke's model. Lucey's work provides a way to calculate *k* of a mixture with inverse Hapke's model. Therefore, the reflection spectrum of the LSCC data can be used to obtain the optical constants (*n*, *k*) of the samples. With the optical constants, radiance coefficient of any phase angle can be calculated via Hapke's model.  Figure 3 shows the relationship between optical constants and reflectance via Hapke's model and inverse Hapke's model.

## 3. Definitions and Assumptions for the Lunar Soil

Unlike the Earth, the density of the atmosphere surrounding the Moon is tenuous. Thus the Moon can be considered a vacuum environment. It is assumed that there is no reflection by the atmosphere. The view of the lunar landscape is described as “the world of rocks.” The only two categories of the compositions of the crust found on the lunar surface are the highlands: Lighter and Anorthositic Surface, and the lunar maria: Darker and Basaltic Planes. Before we explain our method, the model representing the Moon's environment will be defined and several definitions and assumptions will be made in the following subsections.

### 3.1. Refraction/Reflection of the Lunar Soil

**Figure 4:**
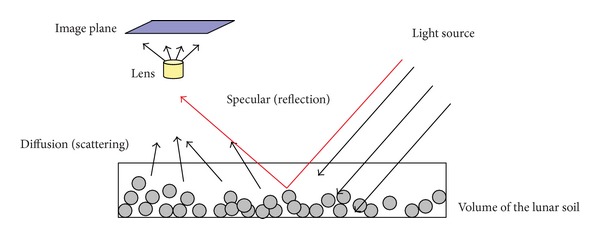
Reflection and scattering of the lunar surface.

Figure 4 shows the reflected rays of the lunar surface—scattering (diffusion) and the specular reflection. The reflection depends on the following.

- Direction of the light source;
- Shape of particles;
- Topology of the surface;
- Compositions of the soil.

Unless there is a large shinny area, the contribution of specular reflection is negligible in a large-scale landscape [19]. Therefore, our method focuses on the scattering and ignores the specular reflection. This assumption will be considered when we simplify (1) in Section 4.2.

**Figure 5:**
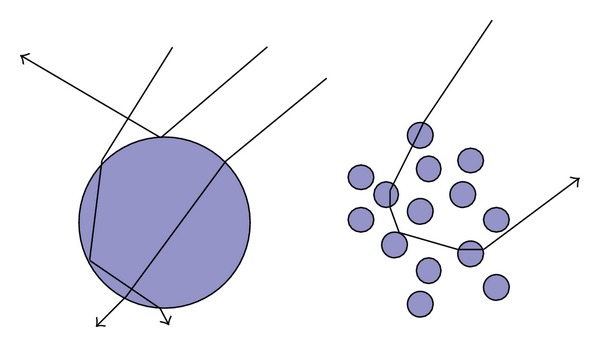
Complexity of refraction/reflection of particles.

When a ray encounters an object, the reflection/refraction can be analyzed using the Snell's Law. Each small bulk of the lunar soil is composed by particles of the mineral. An incident ray travels between these small particles in the soil. Some of the rays (photons) are reflected out of the bulk of lunar soil, while others are refracted inside the mineral and then finally absorbed by the mineral (see Figure 5). Monte Carlo ray tracing of the internal scattering of particles was presented in [1–4]. In the scale of our simulation, computational complexity of such microscale ray tracing is very high as there is a huge number of reflections and refractions. We used Hapke's radiative transfer model instead to represent the intensity of the reflection of a bulk of the lunar soil. If the optical constants are known, then the reflectance of any incident angle and viewing angle (phase angle) can be reproduced. Because we cannot obtain the accurate optical constants, the optical constants of a sample of the lunar soil are estimated using inverse Hapke's model.

### 3.2. Modeling the Lunar Soil

**Figure 6:**
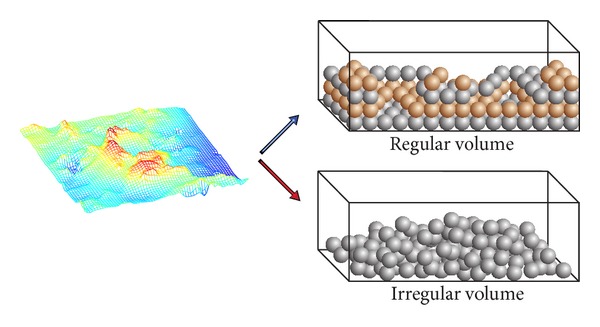
Two types of volume structures representing lunar soils (odd and even layers of voxels of the regular volume are shown in different color for a clear view).

For modeling the lunar soil, a volume is used to represent the topology of the lunar surface. Each voxel (a unit element of a volume) is assumed to be a small bulk of the lunar soil composed by particles such as sands and rocks. Two types of volume structures are shown in Figure 6. Regular volume and irregular volume are proposed in our method. The regular volume is like a regular sampling grid of the lunar soil. It is easy to construct, easy to be implemented and is efficient for a fast simulation. The irregular volume is more precise to represent the position of the actual measured soil samples.

**Figure 7:**
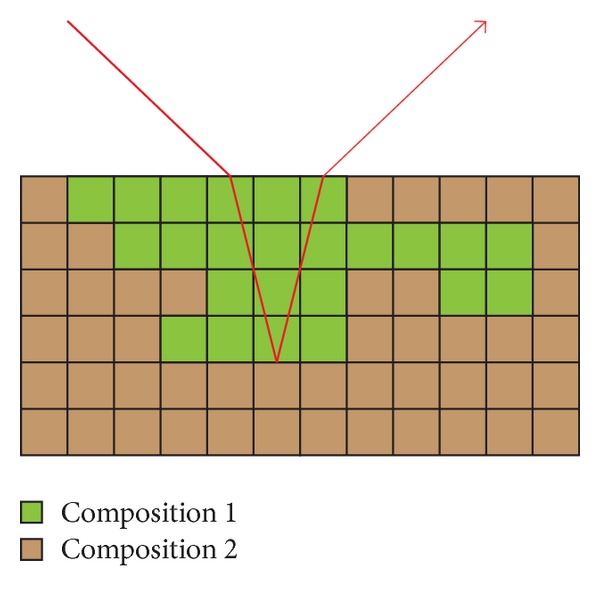
Multilayers reflection model.

Modeling the lunar soil as a volume allows our method to represent the multilayer reflection model of the lunar soil. For example, in a stratified material, if a bulk of the mineral has different composition with respect to the mineral at the surface, a ray penetrates/is refracted through the first layer of the lunar surface, but then is reflected back while it encounters with an internal bulk of different kind of minerals (as shown in Figure 7). Multilayer reflection is much more complex since there will be refraction. Therefore, we would like to leave this aspect as an extension and future work in another paper.

### 3.3. Abstraction of the Model of the Spectrometer

**Figure 8:**
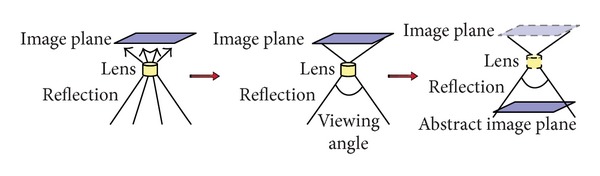
Abstraction of the model of the spectrometer.

In lunar exploration, reflected spectra are measured by the spectrometer installed on an orbiter. We can image that there is a camera installed at the bottom of the spacecraft, taking pictures while the spacecraft flying (orbiting) around the Moon. To simplify it, the camera (spectrometer) can be represented by a lens and an image-plane. Furthermore, since the lens is affected by the viewing angle only (we assume that it will not lose focus), we can use an abstract image-plane and the viewing angle to represent the spectrometer, for producing the measured images of the reflected spectrum. Abstraction of the model of the spectrometer, which captures the reflected spectra of the lunar soil, is shown in Figure 8.

## 4. Our Method and Simulation Steps

Our method implements Monte Carlo ray tracing and Hapke's model. In this section, we will present how we combined Monte Carlo ray tracing and Hapke's model to form the expression that our implementation is based on. Then we will outline the simulation steps.

Monte Carlo ray tracing [20, 21] is known as a well approximation of the solution of the rendering equation [22] which has been investigated and widely applied to global illumination in computer graphics [23–25]. On the other hand, Monte Carlo ray tracing is also widely used in simulating and analyzing the optically contrast structures of particles with complex shapes. Ray tracing describes a method to produce global illumined images of 3D virtual objects; it traces the path and integrates the emitted energy of the light source and reflection between the objects according to the principle of optics. The rendering equation is an integral equation formulated on the definition of the bidirectional reflectance distribution function (BRDF) plus the self-emittance of surface points at light sources as an initialization function. Monte Carlo ray tracing solves this integral equation by Monte Carlo integration.

BRDF and Monte Carlo ray tracing have been applied to remote sensing to investigate the characteristics of the electromagnetic radiation transport between the objects in both macro-scale and microscale, from the surface of the Earth to the internal reflection of particles in a mineral. Our method utilizes Monte Carlo ray tracing to trace the path of the reflected ray between the light source and the encountered object (the lunar surface) and the Hapke's radiative transfer model is used to adjust the intensity of the reflected ray instead of analyzing the internal scattering of the mineral.

### 4.1. Definition of the Scene

**Figure 9:**
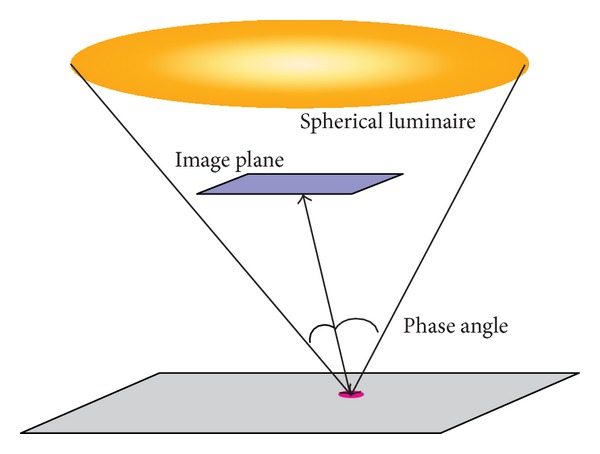
Monte Carlo ray tracing of a spherical luminaire.

From the point of view of Monte Carlo ray tracing, the light source of the scene (the Sun) is a huge spherical luminaire. Each pixel of the measured images is an accumulation of all reflected ray hitting the image plane, emitted from each small part of the luminaire (see Figure 9).

**Figure 10:**
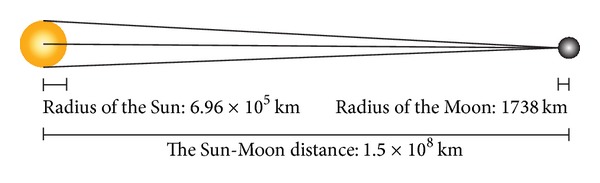
The distance between the Sun and the Moon is much longer than the radius of the Sun.

However, even the radius of the Sun is much longer than that of the Moon (over 400x), the distance between the Sun and the Moon is much longer than the scale of the radius between them (see Figure 10). We can ignore the acreage of the measuring area since it is just a very little piece of the surface of the Moon. The scale of the measuring area is much smaller than the radius of the Moon. Therefore, the measuring area can be represented as a dot in this calculation. The range of the phase angle can be calculated by the following equation:

(2) $$\begin{array}{c} 2\arcsin\left(\frac{\text{Radiu}{\text{s}}_{\text{Sun}}}{\text{Distanc}{\text{e}}_{\text{Sun-Moon}}-\text{Radiu}{\text{s}}_{\text{Moon}}}\right)<0.01. \end{array}$$

**Figure 11:**
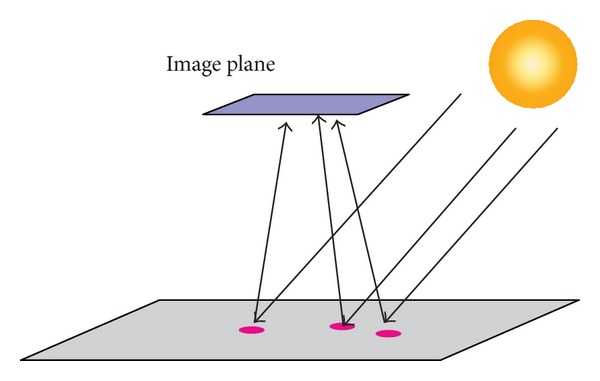
The Sun as a parallel light source.

As the variation of the phase angle is only 0.01 degree, the incident rays can be treated as parallel rays (see Figure 11), so that we can revise the definition of the light source—the Sun is a parallel light source in the scene rather than a spherical luminaire. In our simulation, the range of the phase angle is only depended on the angle between the horizon of the lunar surface and the direction to the Sun.

### 4.2. Reflectance Intensity of the Lunar Surface

Reflection intensity can be determined by the following factors: the direction of the incident light, the position of the viewing point, material, and the shape of the object. The reflection equation [22] we used is listed in the following. Note that the $\vec{\omega }$ here is the direction as of two points; it is not the single-scattering albedos of the Hapke's model:

(3) $$\begin{array}{c} L_{r}\left(\vec{x},{\vec{\omega }}_{r}\right)=\int_{\Omega_{i}}f_{r}\left(\vec{x},{\vec{\omega }}_{i}⟶{\vec{\omega }}_{r}\right)L_{i}\left(\vec{x},{\vec{\omega }}_{i}\right)\cos\theta_{i}\text{d}\omega_{i},\\ \vec{\omega }\left({\vec{x}}_{1},{\vec{x}}_{2}\right)=\vec{\omega }\left({\vec{x}}_{1}⟶{\vec{x}}_{2}\right)=\frac{{\vec{x}}_{2}-{\vec{x}}_{1}}{\left|{\vec{x}}_{2}-{\vec{x}}_{1}\right|}. \end{array}$$

The reflected ray *L*_*r*_ is computed by integrating the incoming ray over a hemisphere centered at a point of the surface and oriented such that its north pole is aligned with the surface normal vector. The incoming radiance along a ray does not change. It obeys the basic laws of geometric optics, assuming that there is no scattering or absorption. BRDF *f*_*r*_ is a probability distribution function describing the probability that an incoming ray of light is scattered in a random outgoing direction.

Then, we can calculate the light intensity from the Phong reflection model [26] as follows:

(4) $$\begin{array}{c} I_{p}=k_{a}i_{a}+\underset{m\in \text{lights}}{\sum }\left(k_{d}\left({\hat{L}}_{m}\cdot \hat{N}\right)i_{m,d}+k_{s}{\left({\hat{R}}_{m}\cdot \hat{V}\right)}^{\alpha }i_{m,s}\right). \end{array}$$

Equation (4) calculates the illumination of each surface point *I*_*p*_, where *k*_*a*_ *i*_*a*_ is the ambient term, the $k_{d}({\hat{L}}_{m}\cdot \hat{N})i_{m,d}$ is the diffuse term, and the $k_{s}{\left({\hat{R}}_{m}\cdot \hat{V}\right)}^{\alpha }i_{m,s}$ is the specular term of the incoming light. Light is defined as the set of all light sources, ${\hat{L}}_{m}$ is the direction vector from the point on the surface toward each light source (*m* specifies the light source), $\hat{N}$ is the normal at this point on the surface, ${\hat{R}}_{m}$ is the direction that a total reflection ray of light would take from this point on the surface, $\hat{V}$ is the direction pointing towards the viewer, and *α* is a shininess constant for this material, which is larger for surfaces that are smoother and more mirror-like. When this constant is large the specular highlight is small.

Specular reflection is ignored in our simulation. Ambient term accounted for the small amount of light that is scattered about the entire scene is also ignored. Then only the diffuse term is remained. Instead of using a diffuse reflection constant *k*_*d*_, we use the radiance coefficient *r*_*c*_ of the equation of Hapke's model (see (1)) to represent the ratio of the mineral in different phase angles. Then, we derive our equation in which our method is based on as follows:

(5) $$\begin{array}{c} I_{p}=\underset{m\in \text{lights}}{\sum }\left(r_{c}\left({\hat{L}}_{m}\cdot \hat{N}\right)i_{m,d}\right). \end{array}$$

### 4.3. Simulation Steps

**Figure 12:**
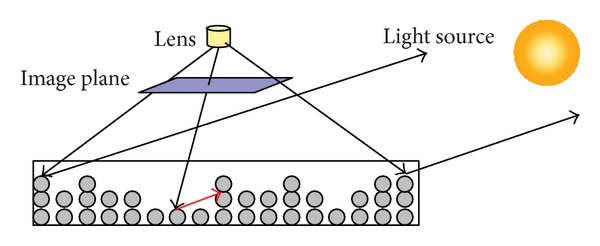
The simulation process.

The simulation result of (5) contains two parts: the Hapke's BDRF contributes the reflectance intensity of the mineral and the diffuse term of the Phong reflection model contributes the reflectance intensity of the topography.  Figure 12 shows the simulation process. To find out the contribution of the mineral of the lunar surface, a path tracing will be processed, starting from each pixel of the image plane. This part is obtained with the following steps.

1. From the position of a pixel of the image plane, trace along a ray to the measured voxel of the volume of the lunar soil, store the position of the measured point and the corresponding mineral.
2. Starting from the encountered point (voxel), trace back to the Sun along the direction of the incident ray.
3. If the incident ray is blocked by other voxel, set the result of the pixel to zero, skips the following steps.
4. Else calculate the phase angle, using the phase angle to find out the reflectance spectrum with Hapke's model.

The reflection of the topography is the diffuse term of the Phong reflection model without the *k*_*d*_—only (${\hat{L}}_{m}\cdot \hat{N}$). This part is calculated with the following steps.

1. Calculate the normalized gradient of the lunar soil (the volume or the elevation map) and use as the normal vector of the surface.
2. For each measured point—the encountered voxel found at the path tracing process—dot products the corresponding incident ray direct and normal vector (both are normalized) to find out the diffusion of the lunar surface.

The specular reflection can also be calculated but we ignored it. The specular reflection should be considered in the simulations of a very small scale landscape with a very high resolution image plane or in the case the lunar surface is very smooth even if there are both mare and highlands exist in the measured area.

## 5. Simulation of the Surface around Apollo 16's Landing-Site

Our method can simulate the reflectance spectrum of several layers of different mineral composition of the lunar soil. However, we do not have such mineral data. Instead, we used Chang'E-1 elevation map [27] and the LSCC data to demonstrate how our method can be used to model the reflectance of the lunar regolith. Chang'E-1 (CE-1) [28] is a satellite operated in a circular polar orbit about 200 km above the lunar surface. The LSCC data (http://web.utk.edu/~pgi/data.html) includes 9 mare and 10 highland samples, which is the only complete “ground truth” data of the Moon consisting of both soil reflectance spectra and mineral abundances. The reflectance spectra were measured in RELAB (http://www.planetary.brown.edu/relab/) at Brown University.

To represent the topography and the mineral of an area of the lunar surface, reflection data of 6 samples of Apollo 16 from the LSCC data and CE-1 elevation map were used. The polar angle and the azimuth angle of the incident light were both set to 45 degrees. The phase angle between the incident light and the reflected light was calculated and then the results were obtained using the reflectance spectrum of Hapke's model of the mineral.

According to the LSCC data, the differences of the reflectance spectrum between the samples of Apollo 16 is bigger than those in other samples. For example, in the visible spectrum, the biggest difference is 0.15 at the wavelength of 330 nm and is 1.9 at 780 nm, where other samples is about 0.5 at 330 nm and less than 0.1 at 780 nm. By using the samples of Apollo 16, we can show the effect of the mineral to the reflection clearly.

The reflectance spectrum of the samples from Apollo 16 project was used, and the topography of the lunar soil volume was reproduced by the laser altimeter of CE-1 of the area around the Apollo 16 spacecraft landing-site (from 9.9375 E to 19.9375 E longitude, 5.0625 S to 15.0625 S latitude). The resolution of the elevation map [27] is 256 × 256.

**Figure 13:**
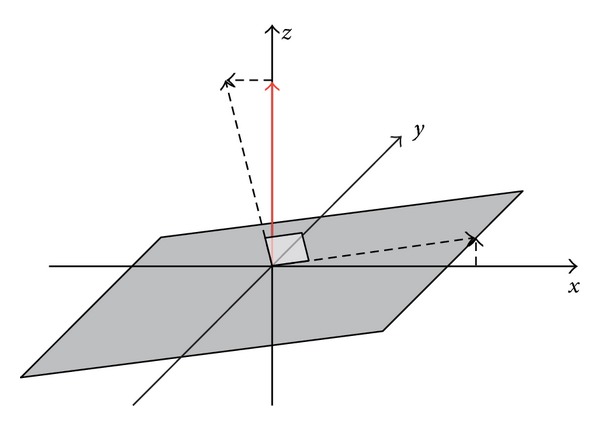
The setup of the topography of CE-1 elevation map.

**Figure 14:**
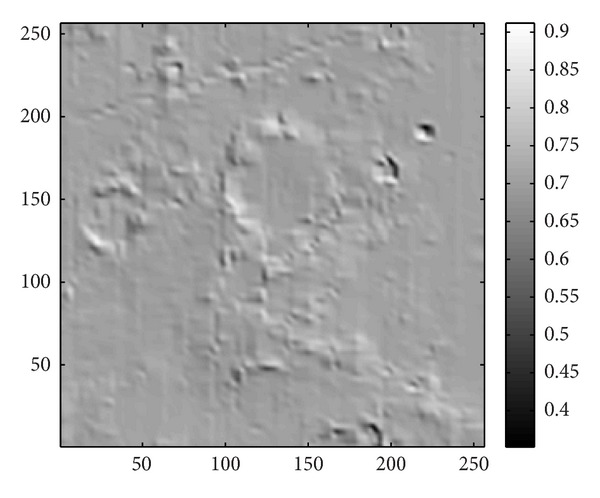
Simulation result of the terrain with diffusion (size: 256 × 256).

The diffusion of the topography was calculated using the normal vectors of the surface. Normal vectors can be calculated using the elevation map. It is a common way to initialize a unit vector pointing along the *z* axis (we assumed that the surface is on the *x*-*y* plane and *z* direction points to the upward), define its length (the unit length) with the same scale as the scale of the *x*-*y* resolution of the elevation map. Then the surface normal vector can be found out by the unit vector minus the gradient of *x* dimension and *y* dimension of the elevation map. The setup of the topography of CE-1 elevation map are shown in Figure 13. The simulation result of the terrain with diffusion is shown in Figure 14. Note that the normal vectors must be normalized before using in the calculation of the diffusion.

In the following subsections, simulation results with different distributions of the mineral will be shown. Testing parameters are set as follows: the spectrometer was set at the height of 10,000 km height, facing to the center of the area, and the image plane is parallel to the lunar surface. The incident angle and azimuth angle of the Sun were both set to 45 degrees. The reflectance of the wavelength *λ* = 645 nm was tested in the simulation.

### 5.1. Uniform Distribution

**Figure 15:**
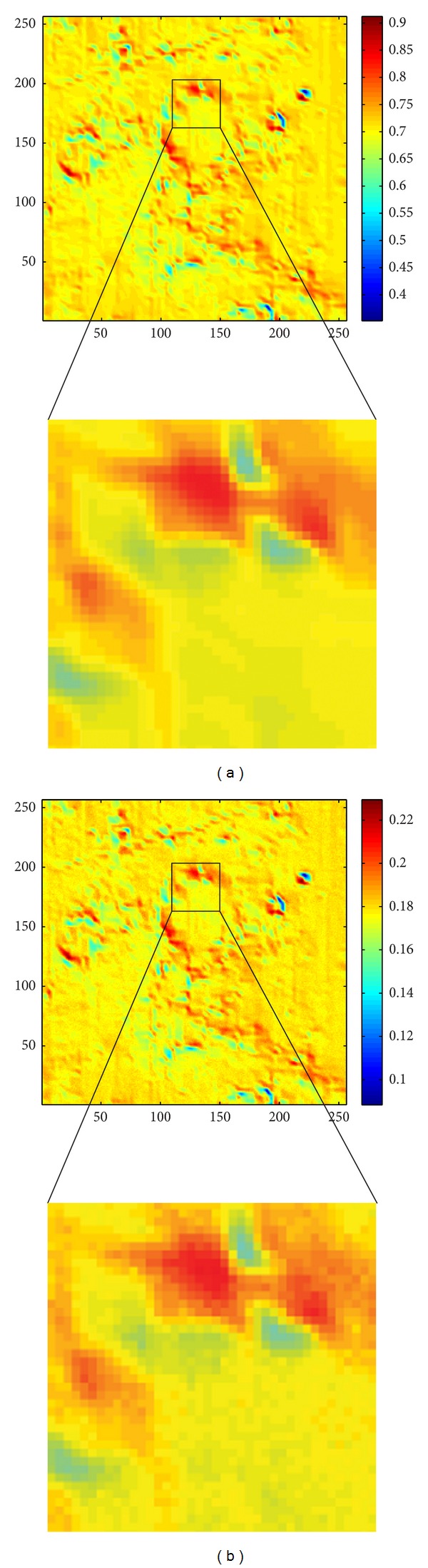
Simulation results of the uniform distribution of the samples (size: 256 × 256). (a) diffusion only; (b) diffusion and reflectance of the samples (sample rate = 2,000).

According to the LSCC data, there are 4 kinds of particle size of each powdered sample. Even for the same mineral composition, samples with different particle size provide different spectra. Therefore, we actually got 6 × 4 = 24 minerals (different spectra) of the Apollo 16 samples. This test case assumed that those 24 kinds of the mineral distributed in the area uniformly (with same probability) everywhere. The simulation result of different sampling rates is shown in Figure 15. From this figure we can realize that the result of the terrain with diffusion only is smooth and the result with the reflectance of the difference of the samples shows the “texture” of the lunar soil.

### 5.2. Location Based Distribution

**Figure 16:**
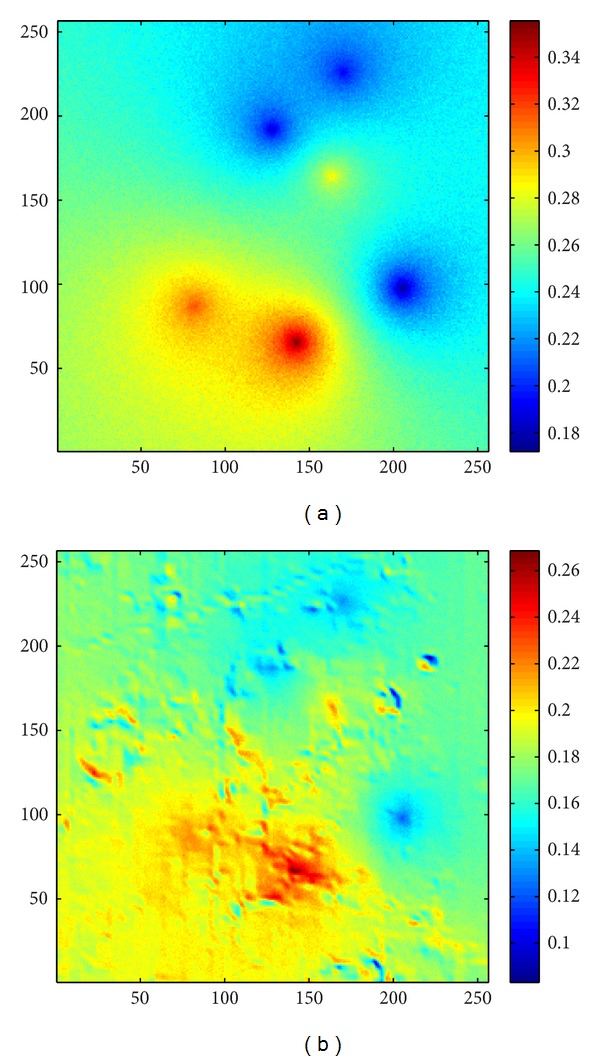
Simulation results of the location based distribution of the minerals (sample rate = 1000, size: 256 × 256). (a) the reflectance of the distribution of the samples; (b) final result of the terrain with diffusion.

In this simulation, 6 kinds of the spectrum of the samples were placed in 6 locations in the area. During sampling, each point of the surface produced reflection intensity with the probability of which kind of the mineral it is. The probability is based on the distance of between that point and the six locations of the samples. Just like when we found a mineral in one specific location, then we considered that the near-by region might contain a similar mineral as well. After the mineral had been determined, the particle size of that kind of mineral was chosen randomly. The simulation result is shown in Figure 16.

### 5.3. Analysis of the Simulation Results

Figures 15 and 16 show the images of a scene of an orbiter that measuring the Apollo 16 landing-site were reproduced by our simulations. The simulation results were affected by the following factors/parameters.

- The position of the luminaire—incident light angle.
- The position of the orbiter—reflection angle.
- The mineral of the volume—reflectance spectrum calculated from Hapke's model.
- The topology of the volume—diffusion and the blocked rays.

From Figures 15 and 16, we can realize that if the diffusion and the reflectance from the radiative transfer model were not considered, we could only produce a smooth surface. If only the reflectance from the radiative transfer model were considered, we could only produce an image with the darker and lighter regions. Such image was not able to show any texture (topography details) of the surface. Once both the diffusion and the reflectance from the radiative transfer model are combined, the reflectance of the rocky lunar surface is revealed. The simulation results with single wavelength (*λ* = 645 nm) are shown in this paper, but our method is able to generate the results with multiple wavelengths.

**Figure 17:**
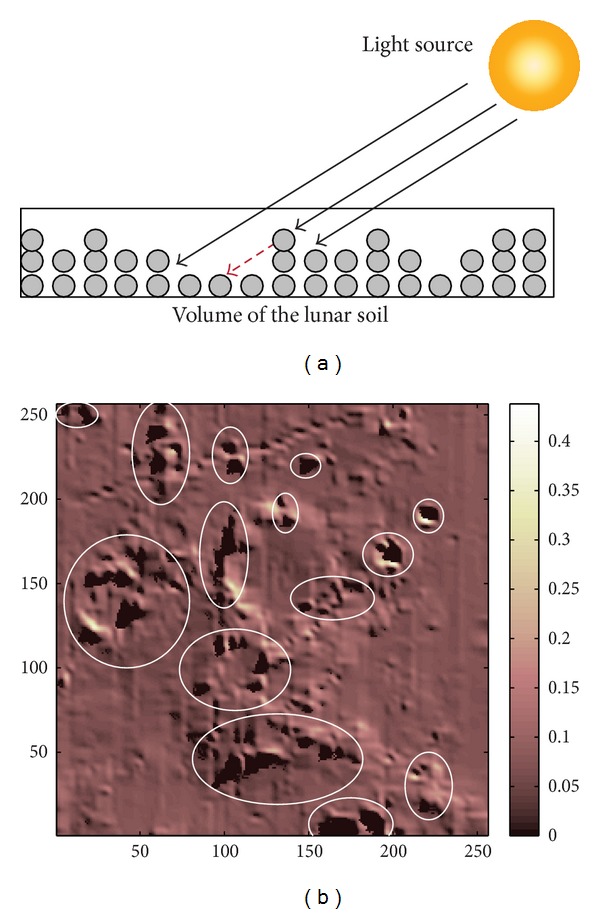
Simulation result with shadows caused by the blocked ray (size: 256 × 256).

Due to the large scale of the width, length, and height of the scene and the position of the Sun, there is no shadow shown in the simulation results (Figures 15 and 16).  Figure 17 demonstrates a scene which shadows are considered when the incident light comes along a big incident angle. Blocked incident lights will produce a zero reflectance to the spectrometer. Shadows and blocked incident lights are not only for generating a realistic reflectance image, but also in analyzing the measured data as we can determine which dark parts were caused by low reflectance rate of the lunar soil or by block incident lights.

**Figure 18:**
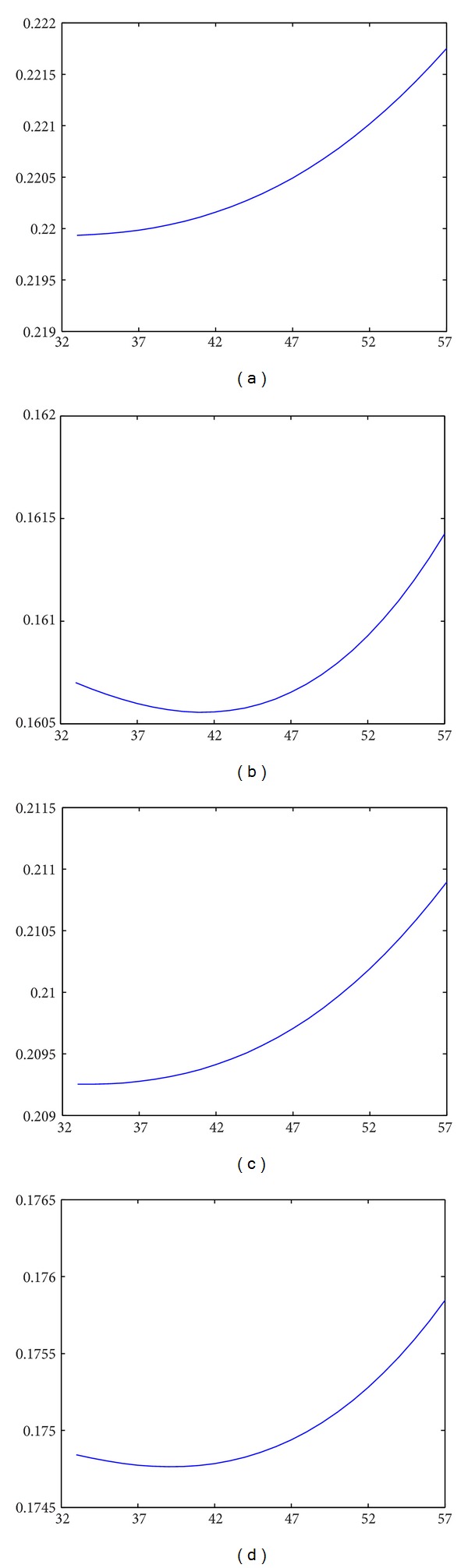
The phase angle from 32.9 degrees to 57.0 degrees of the spectrum of the “61,141” sample (*x*-axis is reduced reflectances and *y*-axis is phase angle (degrees)).

In all the simulation results shown in Figures 15 and 16, the phase angle was varying from 32.9 degrees to 57.0 degrees. According to the results of the Hapke's model, the reflectance should be varying with different phase angles. However, it dose not show the differences very clearly in these results. In our analysis, we found that the reflectance were actually varying but with the order of 10^−3^ (see Figure 18 for the varying of the reflectance index of a simulation result with the “61141” sample). Thus, the varying of the reflectance is too less to be noticed in the resulting images in Figures 15 and 16.

## 6. Applications

In this section, we will show two example applications of our method. The first one is on how to use our method to remove the reflection of the topography in Chang'E-1 IIM data. The reflection will confuse the further analysis of the data. The second one is on how to generate more clear visualizations of the topography of the lunar soil.

### 6.1. Removal of the Reflection of the Topography in Chang'E-1 IIM Data

**Figure 19:**
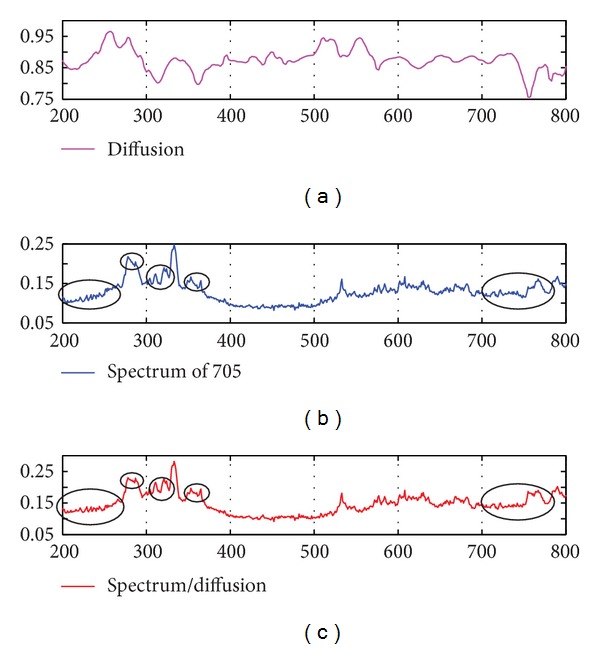
The 1D results of removing the influence of the topography applying our model (in all figures, *x*-axis is wavelength (nm) and *y*-axix is the reflectance). (a) simulated diffusion results; (b) *y*-profile of CE-1 IIM data (*λ* = 705 nm); (c) the result using the inverse model of our method to CE-1 IIM data. The featuring parts to show the changes after dividing the diffusion are circled.

**Figure 20:**
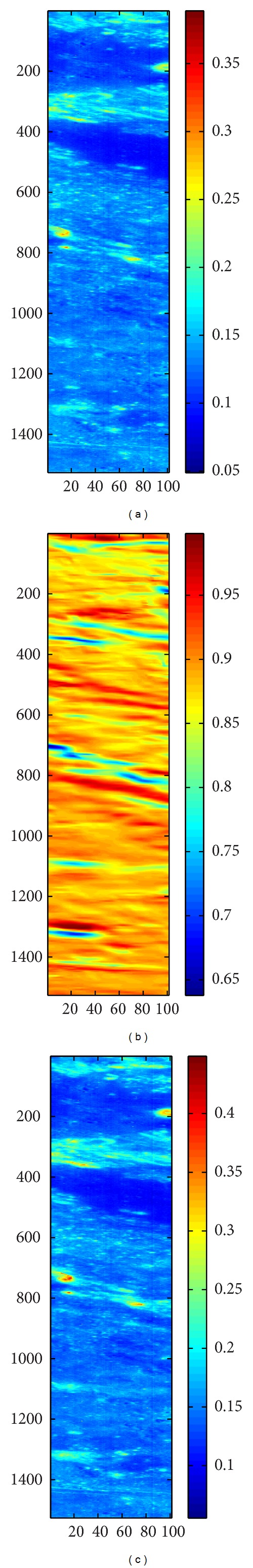
The 2D results of removing the influence of the topography applying our model. (a) *x*-*y* profile of CE-1 IIM data (*λ* = 705 nm); (b) diffusion intensity calculated by CE-1 elevation map of the same region; (c) result obtained by using the inverse process of our method to CE-1 IIM data.

Here, we apply our method to Chang'E-1 IIM data (http://moon.bao.ac.cn/). IIM data measured by the spectrometer contain the impacts from the mineral composition and the shape of the topography. In developing our method, both the reflectance of the topography and the mineral were considered. Therefore, an inverse process of the simulation can be used to remove the reflection of the topography from the raw measured spectra. According to (5) of our method, we can obtain the approximation of the spectrum excluding the effect from the topography by the following formulas:

(6) $$\begin{array}{c} \underset{m\in \text{lights}}{\sum }r_{c,m}=\underset{m\in \text{lights}}{\sum }\frac{I_{p,m}}{\left({\hat{L}}_{m}\cdot \hat{N}\right)i_{m,d}},\\ r_{c,m}=\frac{I_{p,m}}{w_{m}\left({\hat{L}}_{m}\cdot \hat{N}\right)i_{m,d}}, \end{array}$$

where *w*_*m*_ is the weighting factor/function to adjust the effect rate of the topography. As an example, let us take a look to a lunar surface spectrum measured by CE-1.  Figure 19 shows a track of the lunar surface spectrum (*λ* = 705 nm) within the longitude 15.8018 E to 16.4707 E and latitude 5.0619 S to 15.0495 S, the diffusion of our simulation and the result of the spectrum divided by the diffusion. The diffusion of each point was calculated with the same incident angle and azimuth angle as the time while the orbiter was measuring the spectrum. Without considering the noise and other reasons that may course the variation of the measured, we only divided the measured data with the diffusion (*w*_*m*_ = 1). Then, we obtained the results shown in Figure 19. It can be noticed that some slopes and peaks of the measured data were adjusted to horizontal lines. It is probable that minerals at those regions have similar reflectance as their neighboring minerals. And they are possible the same kind of minerals. However, the intensity of the reflected light is different since they were lying on inclines. Therefore, an inverse process of our method can be useful in removing the influence of lunar topography to the reflectance of the lunar soil. The 2D results (*x*-*y* profile) of the same region is shown in Figure 20.

### 6.2. Visualization of the Lunar Surface

Visualization is a powerful tool to understand and analyze the characteristics of volume data. For example, 3D imaging of CT (Computed Tomography) data using volume visualization techniques nowadays plays an important role in daily use in hospitals. In this example application we show how better visualization results of the lunar surface can be obtained using the results simulated by our method. In our method, the lunar soil is modeled as a volume. Volumetric ray casting [29] can be applied to the lunar soil volume to generate a 3D view of the lunar surface without constructing meshes. Besides, special visualization effects such as showing the different composition influences of the lunar surface can be provided.

**Figure 21:**
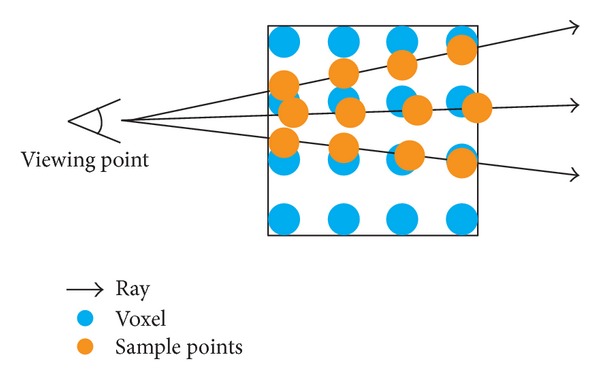
The concept of volumetric ray casting.

Volumetric ray casting [29] is one of the useful visualization algorithms for visualizing volume data, revealing the internal structures of the data.  Figure 21 shows the concept of volumetric ray casting: shooting rays from the viewing point to the volume data, then sampling the voxels along each ray, and compositing the value and with shading to generate the images. For different purpose and visual effects, the calculation of composition and shading can be different. A common way is to apply a user-defined transfer function to map the values of the data to specific RGBA color to reveal the interesting parts of the data. For further information about volumetric ray casting and transfer functions, please refer to [29].

**Figure 22:**
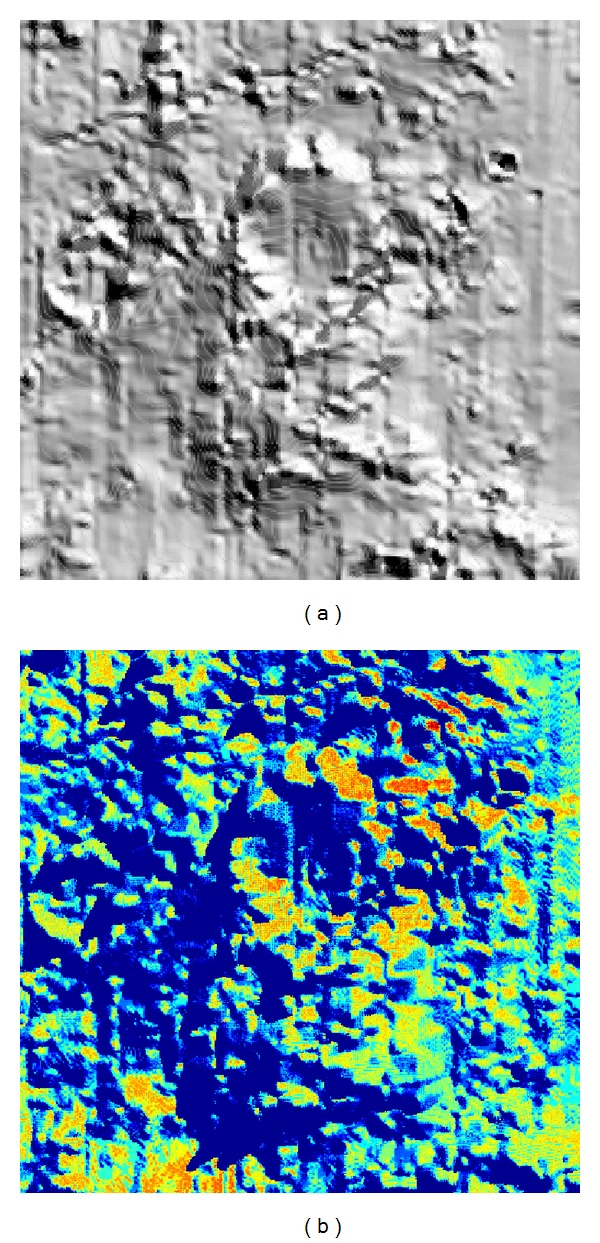
Visualization results of the lunar soil volume. Topography details are enhanced using volumetric ray casting. The result on the right is with shadows and the reflectance of the location based distribution of the minerals.

**Figure 23:**
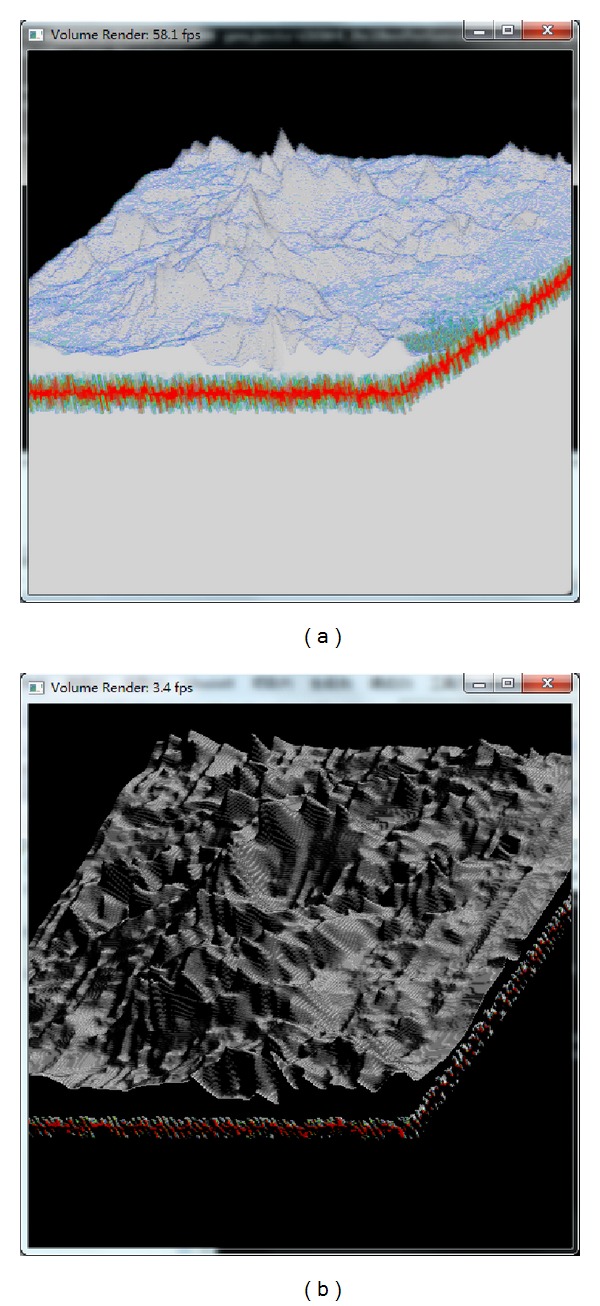
Visualization results of the lunar soil volume. Mineral beneath the surface is generated using a random function. (a) the result without shading effect; (b) the result with shading effect.

Figures 22 and 23 show some visualization results of the lunar soil model. In Figure 23, we simulated if there were internal (multilayer) compositions of the soil. Since we did not have such multilayer information, the composition of the under layer was constructed by a random number generating function. The visualization results shown in Figure 22 were generated by simply adjusting the normal vectors to make the contour of the topography being shown clear, while the results of Figure 23 were generated by mapping with 1D and 2D transfer functions as well as by applying different shading methods to enhance the varying features of topography of the lunar surface.

## 7. Conclusion and Future Work

In this paper, we model the reflectance of the lunar regolith by a new method combining Monte Carlo ray tracing and Hapke's model. Both large-scale effects such as the reflection of topography of the lunar soil and microscale effects such as the reflection intensity of the internal scattering effects of particles of the lunar soil are considered in our method. To the best of the author's knowledge, it is the first attempt to propose a method considering the above effects at the same time, giving a more accurate modeling of the reflectance of the lunar regolith compared to those existing methods using either a radiative transfer model or a geometrical optical model. Our method aims to provide an attempt to apply the calculated optical constants of samples to the scene of the lunar environment, including the factors of the influence of the terrain as well as the light source. As a result, the incident light, terrain, categories of the lunar soil viewing position are all included in our method. Even shadows can be reproduced via the ray tracing algorithm. Phong reflection model was used to calculate the reflection intensity of the topography. The concept of our method follows Monte Carlo ray tracing to find out the path and the phase angle of the reflected rays. Thus, instead of a constant factor, Hapke's radiative transfer model was used to represent the reflection ratio of the mineral. Simulation results of the lunar surface around the Apollo 16 landing-site are shown to demonstrate our method. Reflectance spectrum of the Apollo 16 samples from the LSCC data and the topography data from CE-1 elevation map were used. We also apply our method to Chang'E-1 IIM data for removing the influence of lunar topography to the reflectance of the lunar soil and to generate more clear visualizations of the lunar surface.

We model the lunar soil as volume, then the internal structures or multi-layers structures of the lunar surface can be represented. Therefore, our method can be extended to handle if there is a ray refracted into the soil and then reflected back while the ray encountered a different mineral beneath the lunar surface. In this practice, only CE-1 elevation map was used in modeling the lunar soil. The model of the lunar soil can be adjusted and improved if more measured data of the lunar surface/soil are invoked, such as the slope map and roughness map from the Lunar Reconnaissance Orbiter (LRO) of the lunar surface. We also look forward to applying our method using the data from other lunar exploration projects such as LRO data and KAGUYA data.

Hapke's radiative transfer was used as BDRF in our method to reproduce the reflection index of the mineral. Calculation can only provide approximation results which may contain errors. Therefore, the method can be refined in the future or use another BDRF to obtain more precise simulation results.
